# Hepatic Stellate Cell Modulates the Immune Microenvironment in the Progression of Hepatocellular Carcinoma

**DOI:** 10.3390/ijms231810777

**Published:** 2022-09-15

**Authors:** Pei-Wen Wang, Tung-Yi Lin, Pei-Ming Yang, Chau-Ting Yeh, Tai-Long Pan

**Affiliations:** 1 Department of Medical Research, China Medical University Hospital, China Medical University, Taichung 40447, Taiwan; 2 Department of Traditional Chinese Medicine, Chang Gung Memorial Hospital at Keelung, Keelung 20401, Taiwan; 3 TMU Research Center of Cancer Translational Medicine, Taipei 11042, Taiwan; 4 Graduate Institute of Cancer Biology and Drug Discovery, College of Medical Science and Technology, Taipei Medical University, Taipei 11042, Taiwan; 5 Liver Research Center, Chang Gung Memorial Hospital, Taoyuan 33375, Taiwan; 6 School of Traditional Chinese Medicine, Chang Gung University, Taoyuan 33302, Taiwan; 7 Department of Cosmetic Science, Research Center for Food and Cosmetic Safety, and Research Center for Chinese Herbal Medicine, College of Human Ecology, Chang Gung University of Science and Technology, Taoyuan 33303, Taiwan

**Keywords:** hepatocellular carcinoma, hepatic stellate cells, epithelial-mesenchymal transition, cytokine array, macrophage, network analysis

## Abstract

Hepatocellular carcinoma (HCC) is a major cause of increases in the mortality rate due to cancer that usually develops in patients with liver fibrosis and impaired hepatic immunity. Hepatic stellate cells (HSCs) may directly or indirectly crosstalk with various hepatic cells and subsequently modulate extracellular remodeling, cell invasion, macrophage conversion, and cancer deterioration. In this regard, the tumor microenvironment created by activated HSC plays a critical role in mediating pathogenesis and immune escape during HCC progression. Herein, intermediately differentiated human liver cancer cell line (J5) cells were co-cultured with HSC-conditioned medium (HSC-CM); changes in cell phenotype and cytokine profiles were analyzed to assess the impact of HSCs on the development of hepatoma. The stage of liver fibrosis correlated significantly with tumor grade, and the administration of conditioned medium secreted by activated HSC (aHSC-CM) could induce the expression of N-cadherin, cell migration, and invasive potential, as well as the activity of matrix metalloproteinases in J5 cells, implying that aHSC-CM could trigger the epithelial-mesenchymal transition (EMT). Next, the HSC-CM was further investigated and network analysis indicated that specific cytokines and soluble proteins, such as activin A, released from activated HSCs could remarkably affect the tumor-associated immune microenvironment involved in macrophage polarization, which would, in turn, diminish a host’s immune surveillance and drive hepatoma cells into a more malignant phenotype. Together, our findings provide a novel insight into the integral roles of HSCs to enhance hepatocarcinogenesis through their immune-modulatory properties and suggest that HSC may serve as a potent target for the treatment of advanced HCC.

## 1. Introduction

Hepatocellular carcinoma (HCC) is an significant cause of death due to cancer, which is associated with the increase in the incidences of liver fibrosis/cirrhosis induced by various etiologies, including viral hepatitis, immune disorders, alcoholic cirrhosis, and nonalcoholic steatohepatitis, suggesting that liver fibrogenesis plays a key role in the changes of tumor microenvironment in HCC [1,2,3]. This is predominantly caused by the high risk of chronic hepatic inflammation. Until now, the overall prognosis of patients with HCC remains poor, and the fibrogenic microenvironment, as well as the pathways linked to the progression of HCC, should be urgently addressed.

The development of HCC is modulated by the hepatic microenvironment, which consists of multiple cell types, including hepatocytes, HSCs, and immune cells [4]. Of note, activated HSCs lead to phenotypic changes and increase cell proliferation, while HSCs stimulate the release of cytokines, chemokines, MMPs, and growth factors, which may aggravate liver inflammation and promote HCC progression by inducing EMT [5,6,7,8]. Meanwhile, activated HSCs also release chemokines to recruit immune cells and these immune cells further activate HSCs via cytokine secretion or direct crosstalk between cells [9,10,11]. Among immune cells in the tumor microenvironment, macrophages are the most abundant normal cells. The macrophages, which produce profibrogenic cytokines, have critical roles in liver fibrogenesis and act bidirectionally in the regulation of matrix deposition and resolution. On the other hand, tumor-associated macrophages (TAMs), which may suppress the T cell responses, exhibit protumoral effects, including angiogenesis, tumor cell invasion, and persistent growth [12,13,14]. Therefore, the liver macrophage phenotype is pivotal for HCC initiation [15]. This research implies that the events occurring in the hepatic microenvironment might promote the susceptibility to HCC progression and elicit the prospect of attenuated risk of HCC after successful anti-fibrotic treatment. 

A large number of proteins and molecules are changed both in quantity and quality during the hepatic carcinogenesis [16]; thus, bioinformatic analyses for data mining offers a feasible tool for high-throughput screening and differentially identifying protein targets that are connected to the pathogenesis and pinpointing the signaling pathways, resulting in the deterioration of HCC [17,18]. Herein, we used the MetaCore™ pathway software to comprehensively analyze the differences in protein and cytokine levels for further delineation of cellular interactions and identified associated molecular mechanisms related to the HSC-mediated HCC development.

A pile of evidence has indicated that HSC-derived signaling molecules may consequentially contribute to the recruitment and differentiation of immune cells in the liver, whereas their specific roles linked to the malignant potential of HCC remain to be further explored. Collectively, the present study demonstrated an involvement of activated HSC, HSC-secreted cytokines/molecules, hepatocytes, and macrophages in the development of HCC. 

## 2. Results

### 2.1. The Levels of α-Smooth Muscle Actin (SMA) Implicate the Association between the Degrees of Hepatic Fibrosis and HCC Stage 

**Figure 1:**
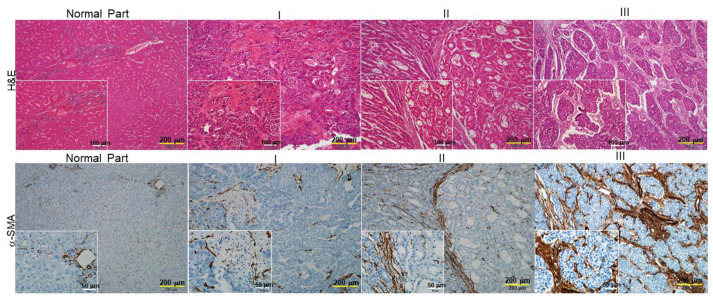
Histological analysis of the association between hepatic fibrosis and HCC stages (I, II, III). Upper panels: Liver fibrosis was determined by hematoxylin and eosin (H&E) staining (bar scale 200 μm and 100 μm). Lower panels: IHC analysis of α-smooth muscle actin (α-SMA) expression in normal and different stages of hepatocellular carcinoma tissues (bar scale 200 μm and 50 μm). The positive signal is indicated in brown and their magnified images are shown in lower right squares. I, II and III indicated stages of HCC.

We first investigated whether liver fibrosis is linked to clinicopathological characteristics of HCC by comparing the status of hepatic fibrosis and the level of α-SMA with various tumor grades. As shown in Figure 1, the hematoxylin-eosin (H&E) staining demonstrated that an intact lobular architecture and normal hepatic cells could be observed in the control sample, whereas the hepatic injuries, manifested as massive necrosis of the hepatocytes; obvious sinusoidal congestion; and inflammatory cell infiltration were worsening with an increasing degree of liver fibrosis. Again, the expression of α-SMA increased significantly in the HCC patients graded as 3, whereas a mild signal of α-SMA was detected in tumor specimens under grade 2, indicating the stage of liver fibrosis could be applied as a valuable indicator for HCC prognosis.

### 2.2. HSC-CM Stimulated Migration and Invasion of J5 Cells under Application of TGF-β1 

**Figure 2:**
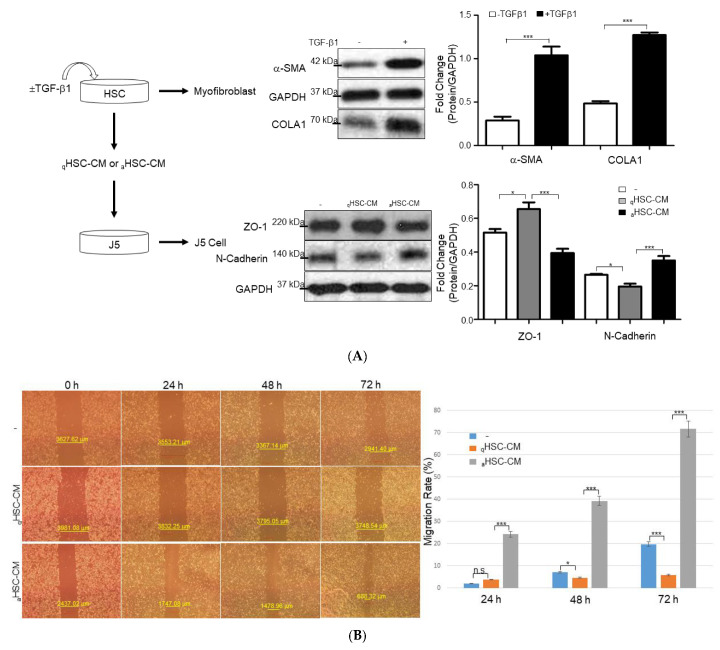

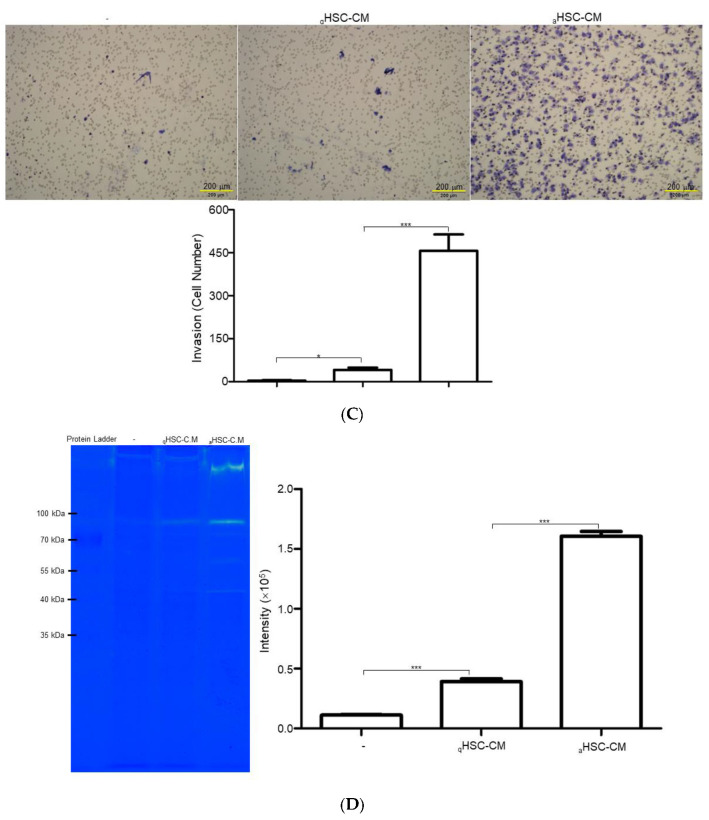
The efficacy of HSC-CM upon HCC J5 cells. (**A**) Validation of the changes of protein levels in HSCs treated with or without TGF-β1 and J5 cells exposed to aHSC-CM or qHSC-CM through Western blot analysis. GAPDH was used as the loading control. The quantified results were indicated by the bar chart (* *p* < 0.05, *** *p* < 0.001). (**B**) aHSC-CM administration enhanced wound closure. Representative phase-contrast micrographs of scratch-wounded confluent cultures with regular DMEM (−), qHSC-CM, and aHSC-CM-treated J5 cells at 0, 24, 48, and 72 h post-wounding (* *p* < 0.05, *** *p* < 0.001, n.s. indicated no significance). (**C**) A marked increase in cell invasion was observed in the human liver cancer J5 cells treated with aHSC-CM, compared to that of regular DMEM (−) and qHSC-CM (bar scale 200 μm). A comparison of the number of transmembrane cells indicates 88% enhancement after the administration of aHSC-CM. The quantified results were demonstrated by the bar chart and represent the mean ± SD of three independent experiments (* *p* < 0.05, *** *p* < 0.001). (**D**) Gelatin zymographic assay was performed using the medium of J5 cells treated with regular DMEM (−), qHSC-CM, and aHSC-CM. The quantitative results were shown as bar charts (*** *p* < 0.001).

To further examine any correlation between particular molecules derived from activated HSCs and the metastatic potential in HCC cells, HSC-CM obtained from HSC-T6 cells treated with or without TGF-β1 (aHSC-CM or qHSC-CM) was applied to J5 cells. At first, HSC-T6 cells were activated with TGF-β1 for 24 hours (h), which was manifested by notably increased protein expressions of α-SMA and collagen I (COLA1), compared with the control group. Western blot analysis also demonstrated that exposure of aHSC-CM to J5 cells significantly increased the expression of N-cadherin, compared to that treated with qHSC-CM, whereas ZO-1 protein was remarkably upregulated in the presence of qHSC-CM, with respect to the sample administrated with aHSC-CM, suggesting that the liver fibrogenic microenvironment could largely affect the EMT of HCC cells (Figure 2A). Next, scratch-wound assays indicated that the aHSC-CM-applied J5 cells migrated particularly rapidly in this assay and filled the empty space within 24 h, but the qHSC-CM-treated cells repopulated the cleared space at a much slower rate within the same time point (Figure 2B). In addition, an 88% increase in the invasive cell number was identified in J5 cells treated with aHSC-CM, compared to that in the control and qHSC-CM-exposed samples (Figure 2C). In line with these results, zymographic experiments indicated that treatment of aHSC-CM significantly promoted the activity of MMPs in J5 cells, while only a trace signal was detected in the control and qHSC-CM-applied samples (Figure 2D).

### 2.3. Characterization of the Changes in Global Cytokine/Chemokine Profile of Different HSC-CM

**Figure 3:**
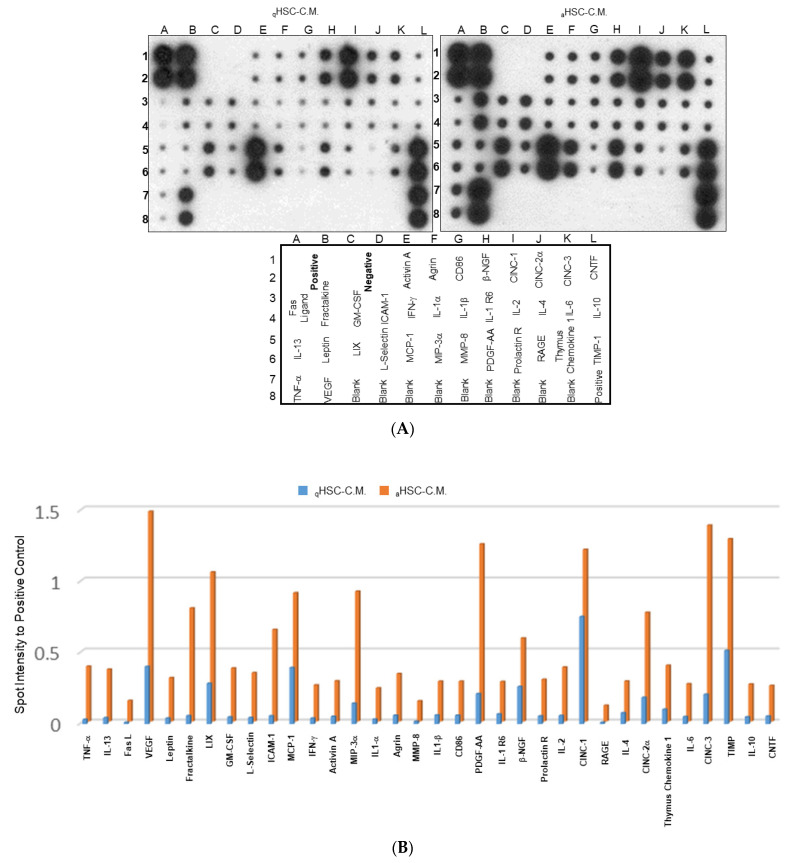

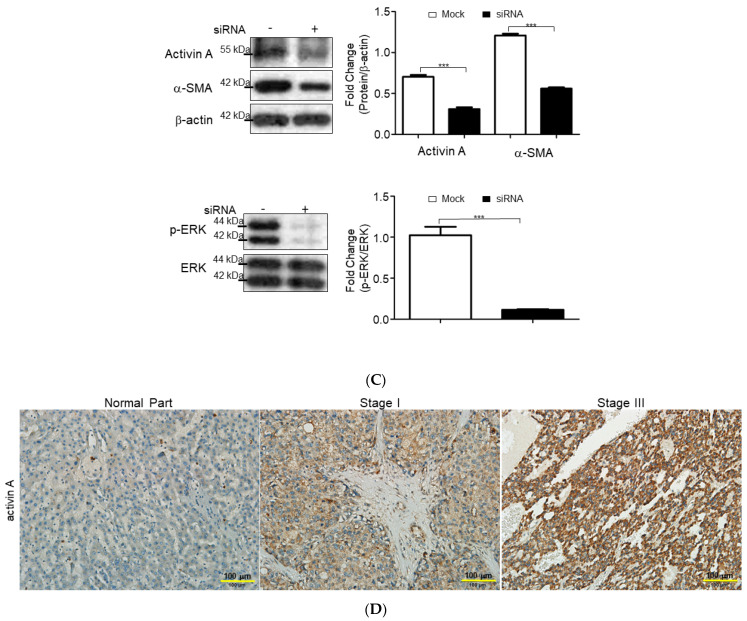
Reveal the overall cytokine/chemokine profile changes by cytokine array. (**A**) Levels of cytokine/chemokine in the aHSC-CM and qHSC-CM were assessed by cytokine arrays. (**B**) The intensity of the chemiluminescent signals for each spot was quantified by GeneTools software. Expression levels were normalized with respect to positive controls on the array membrane. The quantitative results indicate the differences in expression values of cytokine/chemokine and demonstrated as a bar chart. (**C**) HSC-T6 cells were transfected with or without siRNA of activin A. Protein levels were measured by Western blot analysis. β-actin was used as an internal control. The quantified results were indicated by the bar chart (*** *p* < 0.001). (**D**) Immunohistochemical study of activin A expression in adjacent normal part, stage I, and stage III of hepatic cancer tissues. The positive signal was presented in brown (bar scale 100 μm).

The aforementioned results showed that aHSC-CM application might induce the canonical cascades involved in the depravation of HCC. Accordingly, we utilized the cytokine array to delineate the global changes in the expression of cytokines and chemokines that mediated HCC advancement. As demonstrated in Figure 3A, low levels of cytokines and chemokines were detected in the qHSC-CM; several cytokines/chemokines, including the tumor necrosis factor (TNF)-α, IL-13, vascular endothelial growth factor, granulocyte-macrophage colony-stimulating factor (GM-CSF), intercellular adhesion molecule (ICAM)-1, activin A, interferon (IFN)-γ, agrin, interleukin (IL)-1β, CD86, platelet-derived growth factor (PDGF), β-nerve growth factor (β-NGF), cytokine-induced neutrophil chemoattractant-1 (CINC-1), IL-4, IL-6, IL-10, thymus chemokine 1, and tissue inhibitor of metalloproteinase (TIMP), were significantly induced in the aHSC-CM (Figure 3B). Of note, activin A, which is the pluripotent growth and proinflammatory factor of the TGF superfamily was further verified by the functional experiment, utilizing the siRNAs in HSC-T6. The knockdown of activin A obviously suppressed the level of α-SMA, while p-ERK was also significantly arrested with respect to the mock (Figure 3C). The silence of activin A also significantly suppressed the migration and invasion in J5 cells (Supplement Figure S1). In the clinical samples, the expression of activin A were further confirmed, and high levels of activin A were identified in the stage III HCC subject with respect to that of the normal control and lower stage HCC samples, suggesting that activin A could warrant the subsequent malignance of HCC (Figure 3D).

### 2.4. Functional Impact of Activated HSCs on HCC Microenvironment

**Figure 4:**
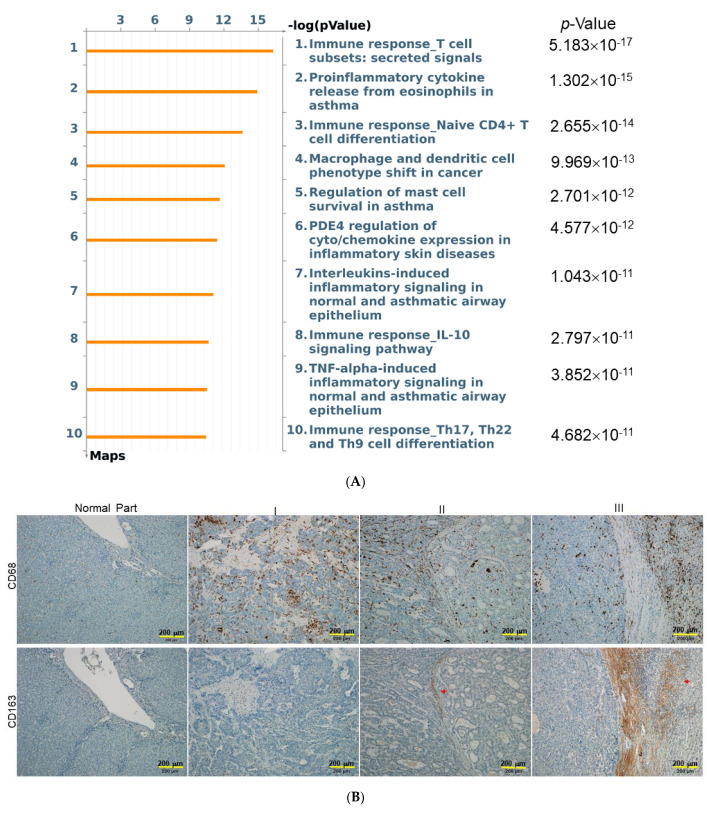

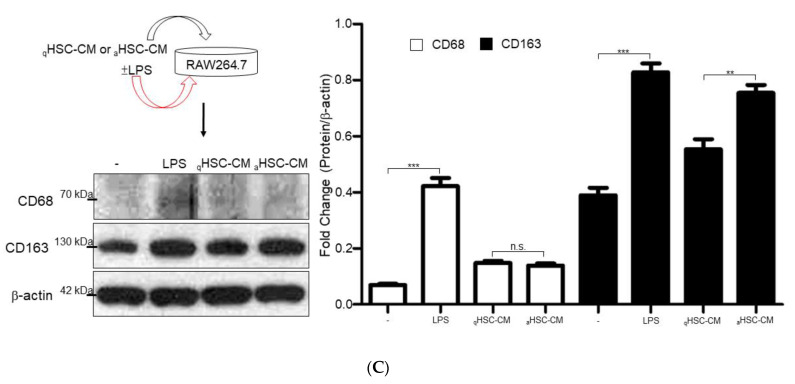
Network analysis to predict the influence of activated HSCs on phenotype shift of macrophages. (**A**) Top-ranked pathways from the GeneGo MetaCore™ pathway analysis are indicated. Pathways were ranked according to p values, and bars represent the inverse log of the *p* values. (**B**) Upper panels: the expression levels and patterns of peritumoral infiltrated CD68^+^ macrophages in different stages of HCC samples. Lower panels: the expression levels of CD163^+^ as indicated by red arrows were significantly increased in the advanced stage of HCC. (bar scale 200 μm) (**C**) RAW264.7 cells were cultured with LPS, aHSC-CM, and qHSC-CM. The expression levels of CD68 and CD163 were determined by Western blotting analysis and β-actin was used as an internal control. The quantitative results were demonstrated as bar charts (** *p* < 0.01, *** *p* < 0.001, n.s. indicated no significance).

To obtain the functional insight of the molecules released from activated HSCs, the underlying pathways regulated by these 34 proteins were built by MetaCore™ software. The MetaCore™ Pathway Maps indicated that these proteins were mainly associated with immune responses, proinflammatory cytokines, and macrophage phenotype shift (Figure 4A). To confirm the above results, the in-situ expression patterns of different macrophage subpopulations was verified by IHC, and a highly expressed M2 macrophage with CD163 marker was found in the tumor and peritumoral liver tissue of stage 2 or 3 HCC samples, compared to that of the normal part and lower grade of HCC. In addition, the cluster of differentiation CD68 may be considered as an M1 macrophage marker. The peri-tumor lesions stained positively for CD68, whereas the majority of tumor cells and hepatic cells stained negatively (Figure 4B). Finally, qHSC-CM and aHSC-CM were applied to RAW264.7 cells to directly observe the macrophage phenotype shift, while LPS was utilized as the positive control. As expected, LPS stimulated the inflammatory response, which manifested as an increase in both CD63 and CD168. The aHSC-CM application significantly promoted the expression of CD168, compared to that treated with qHSC-CM, whereas the level of CD68 showed no obvious difference between samples exposed to qHSC-CM and aHSC-CM, respectively (Figure 4C). These findings indicated that the treatment of aHSC-CM will reprogram the macrophages and activate M2-related genes, which was in congruence with the results obtained from the network analysis.

## 3. Discussion

Hepatic fibrosis is an important risk factor for HCC etiology and the activation of HSCs is the central event of hepatic fibrosis and the development of advanced HCC [19,20,21]. In this regard, the tumor microenvironment built as a result of the interplays among activated HSCs, HCC cells, hepatic ECM, and immune system should be addressed to elucidate the molecular therapeutic targets for preventing liver fibrosis and HCC progression. 

Histopathological examination revealed the positive correlation with the degree of liver fibrogenesis and the severity of HCC, indicating that some molecules released from the activated HSC cells could be applied to stimulate the depravation of the HCC [22]. As expected, aHSC-CM treatment enhanced the metastatic potential of J5 cells, as manifested by the increased ability to invade and migrate. On examining the proteins associated with EMT, we observed that epithelial markers, such as ZO-1, were downregulated, while mesenchymal markers, such as N-cadherin, were upregulated in aHSC-CM-applied cells. Meanwhile, the levels of matrix metalloproteinases (MMPs), including MMP-2 and MMP-9, related with extracellular remodeling and HCC progression were increased under the administration of aHSC-CM [23,24,25]. Therefore, we suggest that activated HSCs release specific molecules to the microenvironment, which enables HCC cell line, such as J5 cell, to acquire invasiveness and metastasis capacity. 

The global cytokine surveys of HSC-CM have demonstrated that TNF-α, agrin, PDGF, activin A, GM-CSF, IFN-γ, IL-1β, IL-4, IL-6, IL-10, IL-13, and TIMP-1, which subsequently induce acute inflammation and liver fibrosis, were elevated in aHSC-CM relative to the qHSC-CM. Of note, TNF-α and IL-13 trigger the activation of quiescent HSCs into myofibroblasts, which stimulate the expression of α-SMA and collagen I, enhancing the accumulation of ECM, as well as the metastatic potential of HCC [26]. The IL-6 level in HCC patients is closely linked to tumor progression and relapse, since IL-6 could induce myeloid-derived suppressor cells to inhibit T-cell immunity and enhance HCC progression [27,28,29]. PDGF is an effective activator of HSC; likewise, we also observed that PDGF expression was elevated in aHSC-CM, which, in turn, induced phenotypic changes in hepatocytes in favor of tumor cell growth. Agrin, a proteoglycan, is a marker of liver tissue because it is most commonly identified in the liver vasculature and in the basement membrane of the bile ducts. It is secreted by activated HSCs in response to PDGF stimulation. Thus, the blockage of PDGF could suppress liver inflammation and fibrosis, as well as inhibiting argin-mediated hepatic carcinogenesis [30]. TIMP-1 secretion was also found to increase in aHSC-CM, which enhances the deposition of ECM to generate the tumor stroma and further abrogate the immune attack. In vitro increases in matrix stiffness have been reported to directly stimulate the growth of the HCC cells and attenuate chemotherapy-induced apoptosis [31]. In particular, activin A is a molecule of the TGF superfamily, implicated in liver fibrosis and cancer [32]. We also confirmed that increased levels of activin A were closely linked to the higher stages in HCC tissue. Moreover, the knockdown of activin A in HSCs and J5 cells showed the roles of activin A in promoting liver fibrosis and malignance of tumor cells. Taken together, the microenvironment created by activated HSCs is prone to induce HCC deterioration via a particular combination of cytokines and chemokines. 

MetaCore™ software was used to elucidate cytokine data. The relative enrichment was responsible mainly for the regulation of immune response and macrophage phenotype shift. Most importantly, great amounts of evidence suggests that macrophages and macrophage-associated factors participate in the development and progression of hepatic fibrosis and HCC [33,34]. Various cellular signals and molecules may trigger the M1-/M2-macrophage polarization. The macrophages undergo classical M1 activation via the stimulation of IL-1β and IFN-γ, while TGF-β1, activin A, IL-4, IL-10 and IL-13 cascades induce alternative M2-macrophages [35,36,37]. CD163 is involved in immune dysregulation, as well as pro-tumorigenesis and it is a confirmed as a specific marker of M2 macrophages. Previous reports have indicated that intratumoral CD163^+^ macrophages are involved in poor prognosis in various cancers [38]. Our results also showed that CD163^+^ macrophages were found to increase significantly within more advanced HCC samples with metastatic potent. On the other hand, the CD68^+^ M1 macrophage is characterized by high level expression of pro-inflammatory cytokines, which modulate the immune microenvironment to trigger liver fibrogenesis [39]. Herein, we observed that the densities of peritumoral infiltrated CD163^+^ cells are positively correlated with expression of CD68^+^ macrophages, demonstrating that the development of liver fibrosis should be closely linked to the advancement and poor prognosis of HCC. We also found that administration of aHSC-CM could stimulate the expression of CD163^+^ and inactivate inflammatory properties manifested as decreases in CD68^+^. As mentioned above, our results imply that activated HSCs build up a microenvironment where HSCs secrete specific cytokine/chemokine and molecules to modulate the immune responses and to shift macrophage subtypes, in turn, resulting in neoplastic progression. 

**Figure 5:**
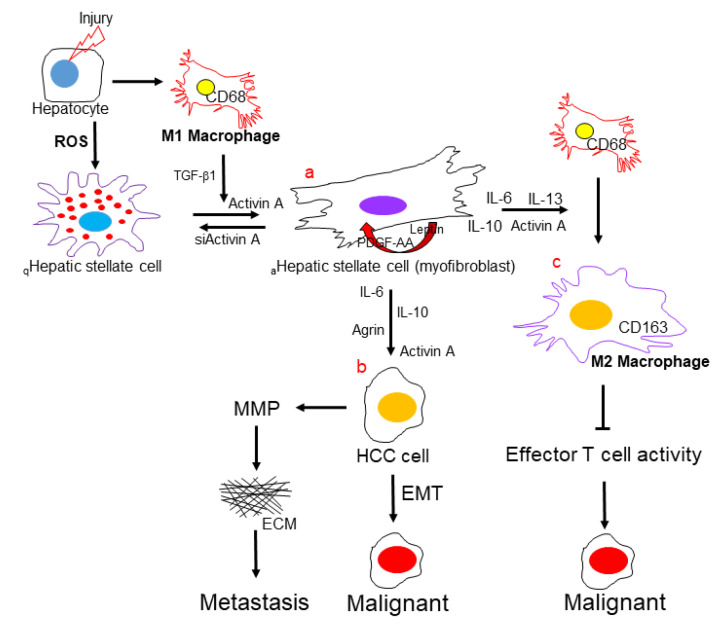
Activated HSCs modulate the immune microenvironment during HCC development. Graphical summary demonstrates the major components, including (**a**) HSCs, (**b**) hepatocytes, (**c**) macrophages, and molecules that are involved in the immune microenvironment in promoting HCC.

In summary, the results of the present study recognized that activated HSCs could enhance the progression of HCC to a great extent via specific pathways, as follows: a. HSC activation during liver fibrosis creates a tumor microenvironment via releasing various cytokines/chemokines or molecules to modulate the immune responses, ECM architecture, and EMT, which plays a central role in the progression of HCC. b. the hepatocytes received the activated HSC-secreted signals, including IL-6, IL-10, agrin, and activin A, which would gain the malignant properties. c. activin A, as a pivotal factor, is closely linked to the shift of macrophage subtypes, which is revealed by network analysis and verified by in vitro experiment. Hence, targeting activin-A could be a novel therapeutic approach to control immune responses and HCC progression. Meanwhile, the incompatible effects of hepatic macrophages can be attributed to their heterogeneous phenotypes and interactions with various nonparenchymal cells and hepatocytes, resulting in the hepatic tumorigenesis (Figure 5).

## 4. Materials and Methods

### 4.1. Tissue Array

We applied commercial tissue array (SuperBioChips Laboratories, Seoul, Korea) to determine the liver morphology, as well as the expression levels of α-SMA, activin A, CD68, and CD163 in clinical samples. These microarray blocks were further applied for H&E and immunohistochemical (IHC) staining. Briefly, the sections were rehydrated with graded ethanol and immersed in Tris-buffered saline after the removal of paraffin with xylene. Next, IHC with antibody in phosphate-buffered saline (PBS) was utilized and sections were counterstained with Mayer’s hematoxylin for 2 min [40]. The slides were observed under a light microscope (Olympus BX51, Tokyo, Japan) and digital photomicrographs were then processed with DP-72 (Olympus). 

### 4.2. Cell Culture

The immortalized rat myofibroblast cell line HSC-T6 was a kind gift from Dr. Scott L. Friedman (Mount Sinai School of Medicine, New York, NY, USA). The HSC-T6 cells were maintained in Waymouth medium containing 10% fetal bovine serum (FBS) at 37 °C in a humidified atmosphere of 5% CO_2_. The human hepatocellular carcinoma J5 cells were cultured with Dulbecco’s modified Eagle’s medium (DMEM) containing 10% FBS for evaluation of HCC cell migration and invasion. RAW264.7, a mouse macrophage cell line, was cultured in RPMI-1640 containing 10% FBS, 100 U/mL penicillin, and 100 μg/mL streptomycin to verify the phenotype shift of macrophages. Experiments were performed with cells after 5~10 passages.

### 4.3. Collection of Conditioning Medium (CM) from HSCs

The rat hepatic stellate cell line, HSC-T6, was cultured in Waymouth medium with 0.2% FBS plus TGF-β1 (5 ng/mL; ProSpec-Tany TechnoGene, Rehovot, Israel). The culture supernatant was collected after 2 days culture as a conditioned medium (aHSC-CM). HSC-T6 cells were subcultured in Waymouth medium with 10% FBS, then transferred into serum free Waymouth medium after attachment, and the supernatant was collected 2 days later (qHSC-CM). The supernatant was centrifuged at 2000 rpm for 10 min to deplete cell debris and then stored at −20 °C. 

### 4.4. Wound-Migration Assay

J5 cells were seeded on a six well plate (1 × 10^5^ cells/well) and cultured with DMEM supplemented with 10% FBS. Twenty-four hours later, the cell layers were scratched with the tip of a pipette and the supernatant was replaced with 2 mL regular medium, aHSC-CM and qHSC-CM. A wound was formed using a 200 μL pipette tip to clear the cell monolayer, and the boundary of the wound was marked. Cells were then washed three times with PBS and incubated for 24 h at 37 °C under a 5% CO_2_ atmosphere. After incubation, cell migration was measured by counting the number of cells that migrated into the clear space using an Olympus microscope (IX71) at 20× fitted with an ocular grid. Results presented are the mean of four random fields of wounds sampled from three independent experiments. The areas of cell migration were determined by dividing the mean number of cells that moved from the edge to the wounded area by cells that moved from the edge in the control culture. The percentage wound area that was filled with proliferated J5 cells for 24 h was calculated as follows: [(mean wound breadth–mean remaining breadth)/mean wound breadth] × 100 (%). 

### 4.5. Invasion Assay

Twenty-four well, 8 μm pore size Matrigel invasion chambers (Corning Inc., Corning, NY, USA) were used for invasion assay. J5 cells were seeded into the upper chamber (5 × 10^4^ cells/well) and cultured with regular DMEM, aHSC-CM and qHSC-CM. Twenty-four hours later, cells that had migrated through the Matrigel were stained with a trypan blue solution (0.4%) and counted, and the invasion index was calculated by dividing the percent of cells that migrated through the Matrigel by the percent of cells that moved through the pores of an uncoated membrane. Invasion was quantified by counting the number of cells in 4 fields per filter at × 100 magnification from three independent experiments.

### 4.6. Gelatin Zymography

Zymographic assays provide a reliable assessment in human cancer progression. J5 cells were cultured with regular DMEM, aHSC-CM, and qHSC-CM and the supernatants were collected. Next, the gelatin zymography was performed as previously described [41]. After electrophoresis, gels were washed with 50 mM Tris–HCl, at pH 7.4, containing 2.5% Triton X-100 (*v*/*v*) for 1 h, then incubated at 37 °C overnight in 50 mM Tris–HCl buffer containing 5 mM CaCl_2_. Digestion was terminated, and gels were stained with 0.5% Coomassie brilliant blue R250 followed by destaining with 10% acetic acid and 10% methanol. Enzyme-digested regions were observed as white bands against a blue background. Zones of enzymatic activity were seen as negatively stained bands.

### 4.7. Cytokine Protein Array and Functional Analysis

The spectrum of cytokines/chemokines in the aHSC-CM and qHSC-CM was determined using an antibody-based protein microarray (RayBio Rat Cytokine C2, RayBiotech Inc.) designed to detect 34 growth factors, cytokines, and chemokines. Proteins were detected via an enhanced chemiluminescence procedure according to previously described procedures [42]. By subtracting the background staining and normalizing to the positive controls on the same membrane, we obtained the relative protein concentrations. To understand the functional role of these 34 growth factors, cytokines, and chemokines, we applied MetaCore™ software (vers. 5.2 build 17389, GeneGo, St. Joseph, MI, USA) to reveal associated ontological classes and relevant pathways. The algorithm builds biological networks from uploaded proteins and assigns a biological process to each network [43].

### 4.8. Western Blot Analysis

Western blot analysis was applied to perform and quantify the amount of protein. The protein obtained from the skin was isolated using 1× cell lysis buffer (Cell Signaling, Danvers, MA, USA) and the concentration was determined with the Bradford Protein Assay Kit (AMRESCO, Solon, OH, USA). The specific antibodies used in the current study were listed as follows: α-SMA (Santa Cruz, Dallas, TX, USA, sc-32251), COLA1 (Santa Cruz, sc-8784), CD68 (Santa Cruz, sc-20060) and GAPDH (Santa Cruz, sc-25778), N-cadherin (Epitomics, Burlingame, CA, USA, 2019-1), ZO-1 (Cell Signaling, 9782), phospho-ERK (Cell Signaling, 9101) and ERK (Cell Signaling, 4695), activin A (myBioSource, San Diego, CA, USA, MBS7103066 & MBS9201920), and CD163 (Bioss, Woburn, MA, USA, bs-2527R). The band intensity was quantified by using GeneTools software (Syngene, Cambridge, UK) and the level of GAPDH was performed as internal control [44]. All experiments were performed in biological triplicate to confirm the reproducibility.

### 4.9. Statistical Analysis

The statistical analysis was executed with Prism software (v5.0, Prism GraphPad, San Diego, CA, USA). The students’ t test was applied for comparison between 2 groups and one-way analysis of variance (ANOVA) was used for multiple groups (≥3 groups) comparison. All data in this study were obtained from at least 3 individual experiments and presented as mean ± standard deviation (SD). The *p*-value <0.05 was considered to be statistically different.

## Supplementary Materials

The supporting information can be downloaded at: https://www.mdpi.com/article/10.3390/ijms231810777/s1.
