# Supplementary material for: Hepatic Stellate Cell Modulates the Immune Microenvironment in the Progression of Hepatocellular Carcinoma
Source: Int J Mol Sci. 2022 Sep 15;23(18):10777. doi: 10.3390/ijms231810777 (PMC9503407; doi:10.3390/ijms231810777)
Supplement: Supplementary file 1 [file ijms-23-10777-s001.zip › ijms-1864547-supplementary.pdf]

Supplement Figure S1

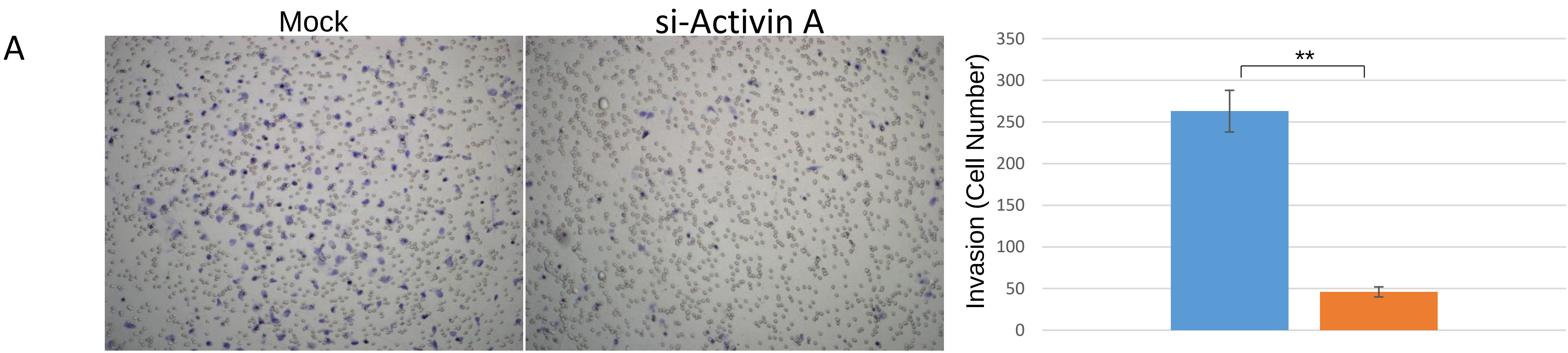


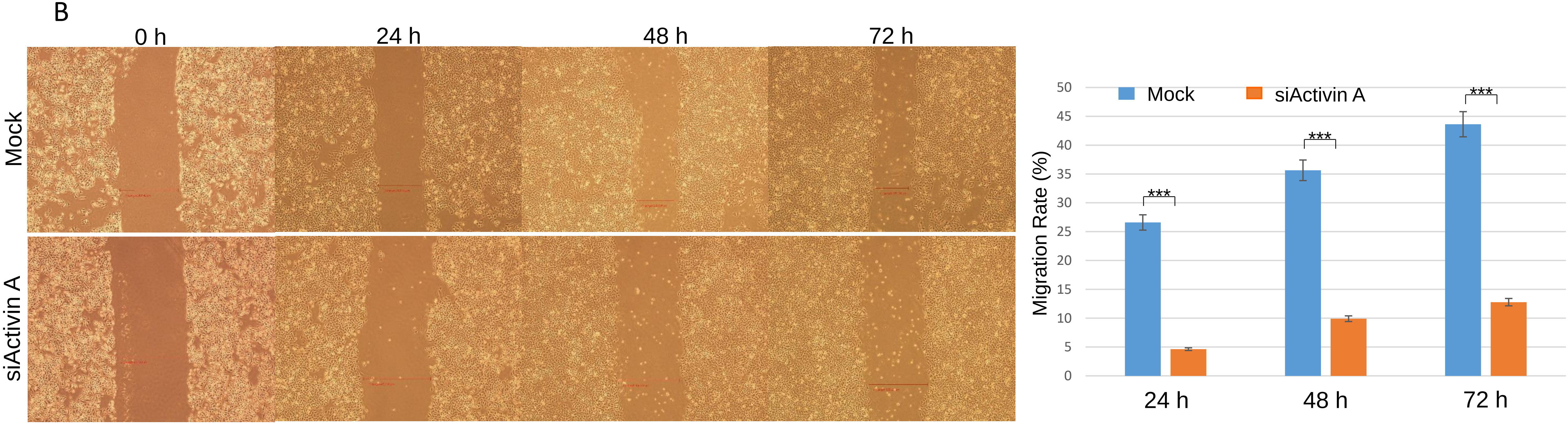


(A) A significant decrease was observed in the invasion of the human liver cancer J5 cells treated with si-Activin A compared to that of mock ($^{**}p <$ 0.01). (B) Representative phase-contrast micrographs of scratch-wounded confluent cultures of mock and si-Activin A-treated J5 cells 0, 24, 48 and 72 h post-wounding ($^{***}p < 0.001$).
